# Integrating artificial intelligence-driven digital pathology and genomics to establish patient-derived organoids as new approach methodologies for drug response in head and neck cancer

**DOI:** 10.1016/j.oraloncology.2025.107742

**Published:** 2025-11-09

**Authors:** Rose Doerfler, Jie Chen, Carl Kim, Joshua D. Smith, Micah Harris, Krishna B. Singh, Brian Isett, Rebekah E. Dadey, Daniel D. Brown, Adrian V. Lee, Xuefeng Wang, Matthew E. Spector, Seungwon Kim, Shaum Sridharan, Kevin Contrera, Katelyn Smith, Carly Reeder, Maureen Lyons, Jianhua Luo, Silvia Liu, Dan P. Zandberg, Heath D. Skinner, Ioannis K. Zervantonakis, Lazar Vujanović, Robert L. Ferris, Raja R. Seethala, José P. Zevallos, Jason J. Luke, Riyue Bao

**Affiliations:** a Hillman Cancer Center, UPMC, Pittsburgh, PA, USA; b Department of Medicine, University of Pittsburgh, Pittsburgh, PA, USA; c Cancer Bioinformatics Core, UPMC, Pittsburgh, PA, USA; d Department of Otolaryngology-Head & Neck Surgery, University of Pittsburgh, Pittsburgh, PA, USA; e Institute for Precision Medicine, UPMC, Pittsburgh, PA, USA; f Department of Pharmacology and Chemical Biology, University of Pittsburgh, Pittsburgh, PA, USA; g Biostatistics and Bioinformatics Program and Immuno-Oncology Program, Moffitt Cancer Center, Tampa, FL, USA; h Department of Immunology, University of Pittsburgh, Pittsburgh, PA, USA; i Department of Pathology, University of Pittsburgh School of Medicine, Pittsburgh, PA, USA; j Organ Pathobiology and Therapeutics Institute, University of Pittsburgh School of Medicine, Pittsburgh, PA, USA; k Department of Radiation Oncology, University of Pittsburgh School of Medicine, Pittsburgh, PA, USA; l Department of Bioengineering, University of Pittsburgh, Pittsburgh, PA, USA; m UNC Lineberger Comprehensive Cancer Center, Chapel Hill, NC, USA; n Division of Malignant Hematology and Medical Oncology, University of Pittsburgh, Pittsburgh, PA, USA

**Keywords:** Head and neck cancer, Organoids, New approach methodologies, Drug response, Artificial intelligence, Genomics, Digital pathology, Human specimens, CNN, Transfer learning

## Abstract

Patient-derived organoids (PDOs) emerge as advanced 3D ex vivo New Approach Methodologies (NAM) pre-clinical models, offering significant advantages over traditional cell lines and monolayer cultures for therapeutic development. In this study, we established PDOs from surgically resected fresh tissues of human papillomavirus (HPV)-negative head and neck squamous cell carcinoma (HNSCC) across anatomical sites, tumor T-categories, and sample types. These PDOs faithfully recapitulate the tumor’s pathology, mutational profile, and drug response. To enable rapid classification of PDO identity, we developed a new convolutional neural network (CNN) model, TransferNet-PDO, which accurately distinguished tumor *versus* normal PDOs in culture using digital histopathology images (AUC ≥ 0.88). PDOs maintained stable cultures and were cryopreserved between passages 5 and 12. Immunohistochemistry (IHC) staining (PanCK, p63, Cytokeratin 13, Ki67) confirmed squamous phenotype and histologic aggression of the original tumor. For tumors harboring TP53 mutations by whole-exome sequencing (WES), PDOs retained the corresponding p53 functional status as confirmed by IHC (enhanced or loss of protein expression). Somatic mutational landscape revealed that PDOs preserved driver somatic mutations, copy number variations (CNVs), and clonal architecture including low-prevalence subclones. Drug sensitivity assessment of PDOs showed that cisplatin reduced cell viability, whereas cetuximab and lenvatinib had minimal effects. Chemoradiation led to greater tumor organoid killing compared to radiation or chemotherapy alone. This study presents an integrated HNSCC PDO platform combining tissue biobanking, organoid establishment, multiomics characterization, functional drug screening, and AI-driven histopathologic classification, providing a comprehensive and scalable system for translational cancer research.

## Introduction

Head and neck squamous cell carcinoma (HNSCC) remains a major clinical challenge, particularly in carcinogen-driven Human Papillomavirus (HPV)-negative HNSCC populations [1]. Despite intensive standard therapies, 40 %–60 % of patients experience disease progression or die within five years. There is an urgent need for improved therapeutic strategies in both the curative-intent locally advanced setting and the recurrent/metastatic setting [2,3].

Murine models have historically been widely used in HNSCC research, providing critical in vivo insights into tumor initiation, progression, and metastasis [4]. However, the translational success remains limited due to fundamental differences between murine and human biology, high costs, lengthy timelines, lack of reproducibility, and low scalability [5]. These challenges have promoted the development of New Approach Methodologies (NAMs) to better recapitulate human biology. Among these, patient-derived organoids (PDOs), three-dimensional (3D) cultures derived from patient tissues, emerge as highly promising for their scalability, genomic fidelity, and translational potential in personalized therapy development [6].

PDOs are well established in cancers such as colorectal [7,8], lung [9,10], and brain [11,12], however models for HNSCC remain relatively underdeveloped. Global efforts are expanding [13–22], however key challenges persist, including overgrowth of normal epithelial or stromal cells, difficulty preserving tumor heterogeneity and clonality over passages, and limited access to fresh surgical specimens. A particularly underexplored area is the ability to reliably distinguish malignant from non-malignant organoids within cultures, which is essential for translational applications. Artificial intelligence (AI), including deep convolutional neural networks (CNNs), has transformed pathology by enabling rapid, quantitative, and precise malignancy detection in digital histopathology [23]. While PDOs recapitulate tumor morphology and genomics, their in vitro growth patterns can pose unique challenges for image interpretation [24]. In this context, AI-driven classifiers offer distinct advantages by detecting subtle single-cell morphologic features, such as nuclear eccentricity or irregular cell borders, across large datasets [25–28]. However, most existing pathology AI tools were trained on primary human tissues [29–35], and their application to organoid systems remains largely unexplored. To our knowledge, no AI-based malignancy classifier has been developed specifically for HNSCC PDOs. Integrating AI into this space therefore presents a timely opportunity to enhance the accuracy, scalability, and translational relevance of organoid models in HNSCC.

**Fig. 1.**
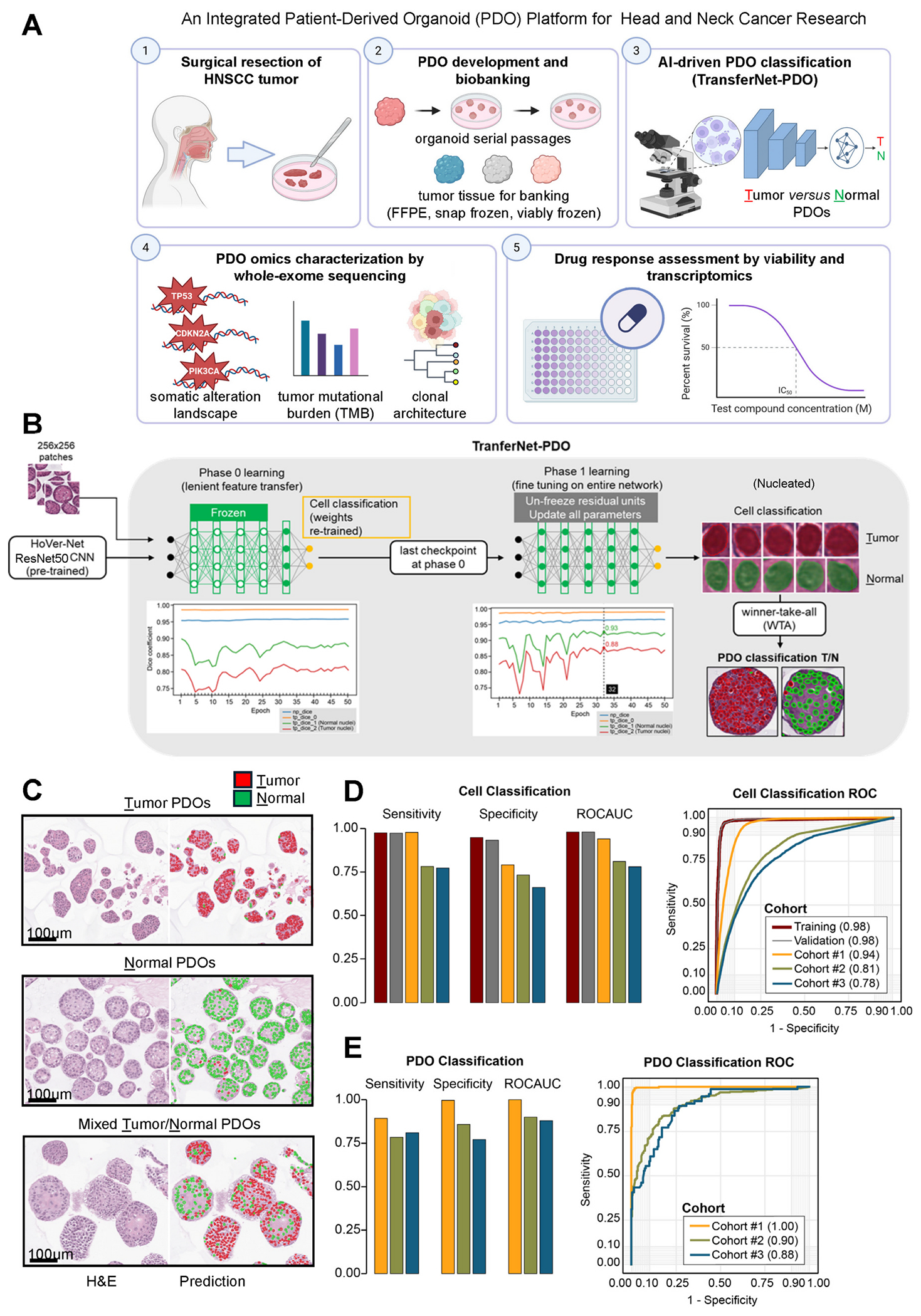
TransferNet-PDO accurately distinguishes malignant from normal PDOs in HNSCC. (**A**) Study design for PDO development and analysis. Five main modules are shown: (1) Fresh tumor specimens from patients with HNSCC are collected at the time of surgical resection; (2) PDOs are developed and expanded through serial passaging; tissue aliquots are banked using multiple preservation methods (FFPE, snap frozen, viably frozen) when available; (3) A new CNN classifier (TransferNet-PDO) is developed to distinguish tumor *versus* normal PDOs from H&E images; (4) PDOs undergo whole-exome sequencing to characterize somatic alterations, tumor mutational burden (TMB), and clonal architecture in comparison to the original tumor; (5) Drug sensitivity is assessed by cell viability assays and transcriptomic analysis. (**B**) TransferNet-PDO deep-learning workflow. 256x256-pixel tiles were extracted from H&E-stained PDO while slide images (WSIs) and input into a pre-trained Hover-Net ResNet50 backbone for nuclear segmentation. In the initial transfer learning phase (Phase 0), classification weights were retrained while backbone features were frozen. In the fine-tuning phase (Phase 1), residual layers of the ResNet50 backbone were unfrozen, and the entire network was optimized using a reduced learning rate. Model performance on segmentation was evaluated using dice coefficients across epochs, with the best checkpoint selected at epoch 32. Final classifications on tumor/normal were made at the single-cell level based on nuclear features, and PDO-level predictions were aggregated using a winner-take-all (WTA; also known as majority voting) strategy. (**C**) Representative examples of classified tumor PDOs, normal PDOs, and mixed tumor/normal PDO cultures. Individual nucleated cells are classified as tumor (red) or normal (green). (**D**) Performance of TransferNet-PDO in classifying individual cells as tumor or normal. (**E**) Performance of PDO-level classification via WTA among classified cells. For **D** and **E**: (**left**) sensitivity, specificity, and ROC-AUC across training, validation, and three independent WSI validation cohorts; (**right**) corresponding ROC curves for each cohort. Detailed methodology is described in Supplementary Methods.

In this study, we address these challenges by establishing a robust cohort of PDOs from fresh HPV-negative HNSCC tumors in patients with diverse clinical characteristics (Fig. 1A). We introduce TransferNet-PDO, a single-cell CNN classifier that accurately distinguishes tumor from normal PDOs using histopathology images. We further show that the clonal architecture of the original tumor is preserved across serial PDO passages, including low-frequency subclones. Finally, we demonstrate clinically relevant drug sensitivities to standard and targeted therapies, highlighting the value of PDOs in preclinical therapeutic testing and precision oncology.

## Methods and materials

The study protocol was approved by The University of Pittsburgh Institutional Review Board (IRB) (IRB99-069). Participants gave informed consent to participate in the study before taking part. All samples have written informed patient consent.

A full description of materials and methods is provided in the Supplementary Methods. In brief, fresh tumor and matched normal tissues were collected via the institutional Head and Neck Cancer SPORE. Tissues were processed to develop PDOs, cultured in Matrigel, monitored by bright-field microscopy, and evaluated histologically. We built TransferNet-PDO leveraging Hover-Net [25] architecture with ResNet50 backbone to distinguish tumor from normal PDOs on H&E images (code: https://github.com/HCC-data-sciences-pub/TransferNet-PDO; model: https://huggingface.co/hillmancancercenterds/TransferNet-PDO). Whole-exome sequencing (WES) was performed to characterize genomic alterations and clonality in tumor and PDOs, with somatic mutations identified by GATK4-MuTect2 [36] (v4.6.1.0), somatic copy number variations (CNVs) by GATK4-somatic CNV, and clonal architecture by RETCHER [37] (accessed January 2025). Drug responses to cisplatin, cetuximab, lenvatinib, radiation, and chemoradiation were assessed using the CellTiter-Glo^®^ luminescent assay, which measures intracellular ATP as a proxy for metabolically active (viable) cells.

## Results

### HNSCC PDO models recapitulate the morphological and pathological features of the original tumor

To establish PDOs from fresh HNSCC tissues, tumor specimens were collected during surgery and transported at 4 °C to the research laboratory (Fig. S1A). Processing occurred within four hours of collection for all samples, and in most cases within two hours. Tumor tissues were minced, digested with trypsin, and embedded in 3D Matrigel for culture (Fig. S1B). Organoids retained epithelial morphology across serial passages and displayed malignant histopathologic features resembling the original tumor per pathology review. Bright-field imaging revealed a typical growth trajectory, from dispersed cells on day 0 to well-formed organoid spheres by day 14 (Fig. S1C). H&E staining and immunohistochemistry (IHC) with HNSCC morphology markers (PanCK, p63, CK13) and cell proliferation marker Ki67 (Table S1) confirmed squamous phenotype and histologic aggression (Fig. S1D). While initial cultures may contain a mixture of epithelial, immune and stromal cells, PDOs progressively became predominantly epithelial, consistent with prior reports [38].

Patient cohort characteristics are summarized in Table 1. After excluding one necrotic sample, tumors from 23 patients contained sufficient viable cells for PDO development (Fig. S1E). Organoid cultures were successfully established in 19 of 23 cases (82 %), yielding predominantly tumor PDOs (n = 14), and normal epithelial organoids overtook the culture in a minority of cases (n = 5). PDOs were derived from a diverse set of tumors spanning anatomical sites (oral cavity, larynx, etc.), T categories (T2/3/4), and sample types (primary, recurrent, second primary), representing patients of different genders, racial backgrounds, and ages (Table 1; Fig. S1E–G). PDOs were established from tissue inputs as small as ~100 mg (median: 366 mg; Fig. S1H). Tumors of all 19 cases were subsequently found to be p53-mutant by IHC staining (Table 1). Notably, p53 status was not used as a selection criterion; all cases with sufficient viable tissue were included, and p53 IHC was performed retrospectively after specimen collection. This observation reflects the high prevalence of *TP53* mutations in HPV-negative HNSCC [39].

Overgrowth of normal epithelial cells presents a frequent challenge in PDO development [40], as also reported in lung [38,41] and prostate [42] cancers. Given tumor tissues often contain a mixture of malignant and normal epithelial cells, the latter can in some cases expand rapidly and outcompete tumor cells in culture. To address this issue, we applied Nutlin-3 [43], an MDM2 inhibitor that activates the p53 pathway and selectively induces apoptosis in wild-type p53 cells (typically normal epithelial cells) (Fig. S2A–B). Nutlin-3 treatment has been used in many types of tumor organoids, including lung [38,44] and liver [45]. In mixed populations, this strategy achieved nearly 100 % malignant cell content post-selection (Fig. S2C). Whereas in other tumor types normal epithelial cells can cease proliferation after multiple passages [46], we observed that in HNSCC cultures, normal epithelial cells overgrew tumor cells as early as passage 1 (p1) and persisted throughout the rest of passages (Fig. S2D). In 6 of 14 tumor PDO cases (43 %), Nutlin-3 was selectively applied at early passages where organoids exhibited normal-like morphology by bright-field imaging (Fig. S2E–F; Table 1), with subsequent WES confirming the absence of driver somatic mutations. These cases were more frequently derived from larger tissue specimens, likely reflecting a greater proportion of normal epithelial cells (Fig. S2F). Together, these results establish a robust and reproducible platform for generating high-fidelity HNSCC PDOs from fresh surgical specimens, setting the foundation for further analyses.

### AI model predicts malignancy in PDO cultures consistent with the original tumor

While PDOs were established from a majority of HNSCCs, distinguishing tumor from normal or normal-like epithelial organoids remains critical for accurate interpretation of drug sensitivity experiments. Considering the relative growth of tumor *versus* normal PDOs varies across patients and specimens, this issue is particularly important in early passages where mixed populations are observed. As an initial benchmark, we examined the distribution of organoid size and shape. Normal PDOs appeared larger on average than tumor PDOs (9,650 ± 299 *versus* 6,657 ± 250 μm, maximum diameter; mean ± S.E.M., n = 17 images); the difference was not statistically significant (*P* = 0.11, linear mixed-effects models [LMM]; Fig. S3A–B). Morphologically, normal PDOs typically exhibited a well-differentiated cystic structure with smooth boundaries, whereas tumor PDOs formed irregular, solid spheres on bright-field imaging (Fig. S2E) and displayed abnormal nuclear features on H&E (Fig. S3C).

To classify PDO identity at scale, we developed TransferNet-PDO, a new framework based on Hover-Net [25] architecture with transfer learning, to predict individual cells from H&E images as tumor or normal, and assign PDO-level predictions by winner-take-all (WTA, also known as, majority voting; Fig. 1B). For training, we generated 256 × 256-pixel tiles of high-density tumor or normal organoids (hereafter referred to as, PDO large-tile dataset) from early passages, segmented nucleated cells using a pre-trained ResNet50 CNN [25], and manually annotated nuclei to build a balanced training set (1,490 tumor nuclei from 88 tiles + 1,545 normal nuclei from 78 tiles). After feature transfer (phase 0) and full model fine-tuning (phase 1), we selected the best-performing model based on ROC-AUC (area under the receiver-operating characteristic curve) (Fig. S4A; Fig. 1B; examples shown in Fig. 1C).

After training, the model was validated on three distinct cohorts of whole-slide images (WSIs; hereafter referred to as, PDO WSI datasets), each designed to assess a different aspect of model generalizability (Fig. S4B). Specifically, we used: (1) non-overlapping regions (~87.5 %) from the same WSIs used to create the PDO large-tile dataset, testing performance on unseen areas within the same images (34,859 cells); (2) WSIs from later passages of the same specimens, testing robustness to temporal variation in PDO morphology (25,625 cells); and (3) WSIs from entirely different patients, testing generalization to unseen individuals (19,570 cells) (Fig. S4B; Fig. S5). At the single-cell level, TransferNet-PDO achieved ROC-AUCs of 0.94, 0.81, and 0.78 on these datasets, respectively (Fig. 1D). At the PDO level, applying a WTA approach to assign each PDO’s class labels yielded ROC-AUCs of 1.00 for the same images, 0.90 within the same patients, and 0.88 across different patients (Fig. 1E). Together, these findings demonstrate that TransferNet-PDO enables accurate, scalable, and robust classification of tumor *versus* normal PDOs directly from H&E slides, offering a practical tool to enhance the reliability of organoid-based research and potential clinical applications.

### Whole-exome sequencing identifies consistent genomic alterations between HNSCC PDO models and the original tumor

To assess the genomic fidelity of PDOs relative to their original tumors, we performed WES on frozen tumor specimens, matched normal tissues, and serial PDO passages (~200 × per sample after duplicate removal), identifying somatic mutations in individual tumor and PDO with normal as control. We focused on WES data from six cases where Nutlin-3 selection was not applied, ensuring that tumor clonality reflected only baseline organoid culture conditions (Table S2).

**Fig. 2.**
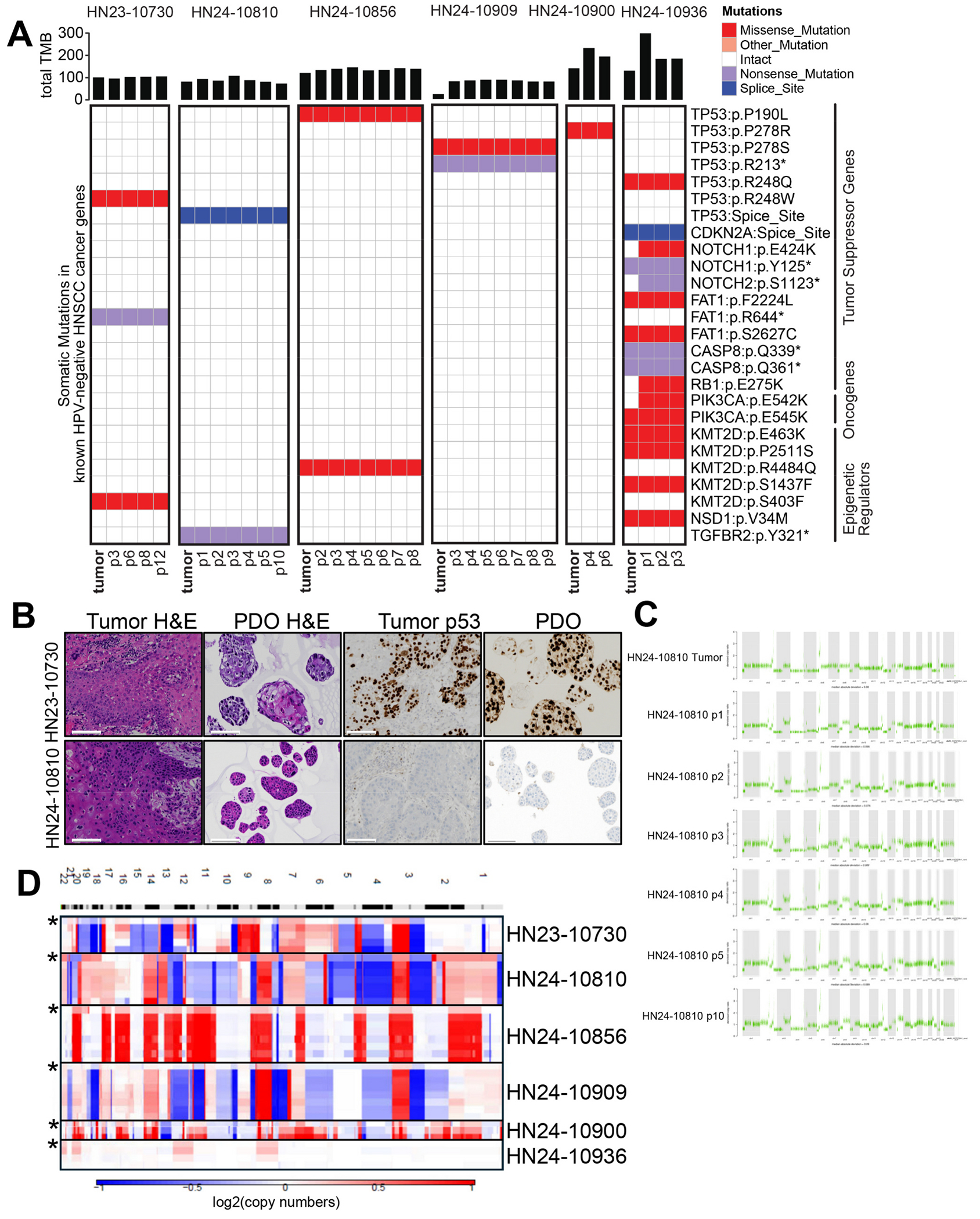
HNSCC PDOs show high-fidelity genomic landscape. (**A**) Somatic mutations in known HNSCC cancer genes across six HPV-negative HNSCC patient tumors and corresponding PDOs across serial passages (indicated as p#). (**top**) bar plots show total tumor mutational burden (TMB) per sample, defined as the number of protein-altering somatic variants (SNVs and small insertions/deletions [indels]) by whole-exome sequencing (WES); (**bottom**) the heatmap displays mutations in known genes involved in HNSCC cancer development and progression, categorized by functional class: tumor suppressors, oncogenes, epigenetic regulators, and others. Each row indicates a unique variant shown to the right of the heatmap; each column represents a tumor or PDO sample. Variant classifications are colorcoded. (**B**) Representative H&E and p53 IHC staining of tumor tissues and matched PDOs from two patients (HN23-10730 with positive p53, and HN24-10810 with negative p53). PDOs show histologic and immunohistochemical concordance with the original tumor. (**C**) Representative example of tumor and serial PDO passages from patient HN24-10810 (**top to bottom**: p1-p10) showing genome-wide somatic copy number variations (CNV). Denoised copy ratio is shown on *y*-axis. Chromosome numbers are shown on *x*-axis. CNV patterns remain largely stable across passages, reflecting genomic fidelity. (**D**) Heatmap of log_2_ copy number changes across the genome for all six patient-PDO pairs from **A**. Each row represents a tumor (denoted by asterisk, first row of each panel) or PDO sample; chromosome number and cytobands are shown on the *x*-axis. Gains (red) and losses (blue) are shown relative to diploid baseline, demonstrating preservation of major CNV events in PDOs.

Tumor mutational burden (TMB) remained consistent between PDO passages and their original tumors (Fig. 2A, bar plot above the heatmap), with minor fluctuations from gain or loss of low-frequency passenger mutations. Driver mutations were stable over time, and most non-overlapping variants represented passenger events, consistent with literature [20]. Across all PDO passages, protein-altering somatic mutations in key driver genes associated with HPV-negative HNSCC [39], such as *TP53, CDKN2A, NSD1, PIK3CA, NOTCH1, FAT1, CASP8*, were consistently retained with minimal disruption relative to the original tumor (Fig. 2A; Table S3; full somatic mutation lists in Table S4). Each patient harbored distinct *TP53* mutations (e.g., P190L, P278R, P278S, P213*, P248Q, R238W), and in HN24-10909, two *TP53* mutations (P278S, P213*) co-existed. In HN24-10909 and HN24-10936, low tumor cellularity in the original specimens likely reduced mutation detection by WES, but these somatic mutations were reliably detected in tumor-derived PDOs. *TP53* mutation status from WES was verified by p53 IHC (Fig. 2B, p53 positive/negative examples shown). In contrast, normal PDOs exhibited wild-type p53 by IHC and lacked recurrent somatic driver mutations. Once tumor cells became dominant, they rapidly expanded and persisted across passages, with *TP53* somatic variant allele fractions approaching 100 % (Fig. S6A). One exception was HN24-10936, where malignant cell populations expanded in PDO over time yet remained mixed with normal epithelial cells. Overall, these molecular findings aligned with our pathological observations, indicating that tumor *versus* normal identity was often established early in culture.

Somatic CNV analysis further supported the genomic stability of PDOs, showing strong concordance between original tumors and PDO passages (Fig. 2C–D; Table S5). While WES data were analyzed for PDOs up to passage 12 (p12), H&E images of later passages (p14) showed pathology comparable to earlier passages (Fig. S6B). Together, these findings demonstrate that HNSCC PDOs faithfully preserve the mutational landscape of the original tumors across serial passages, reinforcing their genomic stability and translational relevance for tumor modeling, drug testing, and precision oncology applications.

### HNSCC PDO models preserve clonality architecture across serial passages

**Fig. 3.**
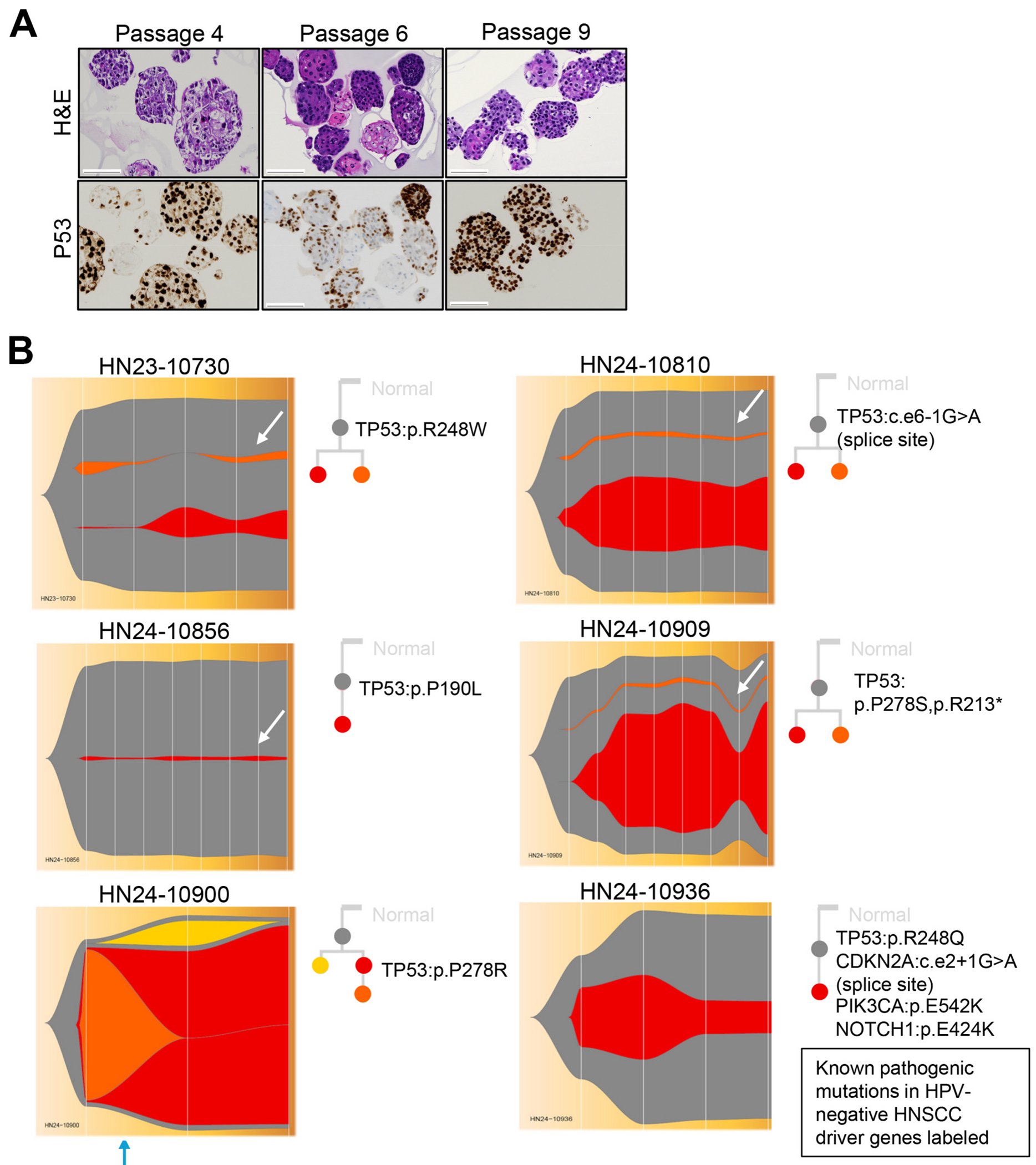
HNSCC PDOs maintain clonal architecture over time. (**A**) Representative example of H&E and p53 IHC staining of PDOs from patient HN23-10730 across passages 4, 6, and 9. PDOs retain malignant histology and consistent nuclear p53 accumulation over time, reflecting the underlying *TP53* mutational status. Scale bar = 100 μm. (**B**) Clonal architecture reconstruction from WES somatic mutation and CNV analysis for six HPV-negative HNSCC cases from Fig. 2. Fishplots depict subclonal dynamics from the original tumor across PDO passages, with each vertical line representing a sample in the same order from Fig. 2A. *TP53* mutant clones were the founding clones in 5 of 6 cases. Case HN24-10900 showed a clonal shift after antifungal treatment (blue arrow). Minor subclones (white arrows) were preserved. Known pathogenic mutations in HNSCC driver genes are annotated next to each plot, with colored dots on the phylogenetic tree corresponding to specific subclones from the fishplots.

Upon confirming consistent p53 mutant status from early to late passages (Fig.3A), we sought to evaluate whether HNSCC PDOs preserve the global clonal architecture of their original tumors. Retention of subclonal diversity is critical for modeling intratumoral heterogeneity and for using PDOs as preclinical tools to study therapeutic response. To address this, we reconstructed subclonal structures using integrated somatic mutation and CNV data (Fig. 3B;Table S6). In five out of six sequenced cases, the founding clone was *TP53*-driven and remained stable across PDO passages (Fig. 3B). In one exception (HN24-10900, oropharynx), *TP53* was detected as a subclone in the original tumor. Organoids of this case required antifungal treatment to eliminate a fungal infection from tumor (Fig. 3B, blue arrow), which altered the clonal dynamics and led to the loss of a minor subclone (Fig. 3B, yellow node on the tree). Despite this, the *TP53* subclone persisted and drove organoid growth, preserving malignancy in culture.

Among the other five cases, we observed evidence of subclonal evolution within the dominant *TP53* clone. For example, in HN24-10936, a *PIK3CA/NOTCH1*-driven subclone emerged and remained stable across passages. Subclones of low prevalence (<5% abundance) were retained in multiple cases (Fig. 3B, white arrows), suggesting that minor subpopulations from the original tumor can be retained in serial PDO cultures.

### HNSCC PDO models demonstrate sensitivity to chemotherapy, radiation, chemoradiation, and targeted therapy

**Fig. 4.**
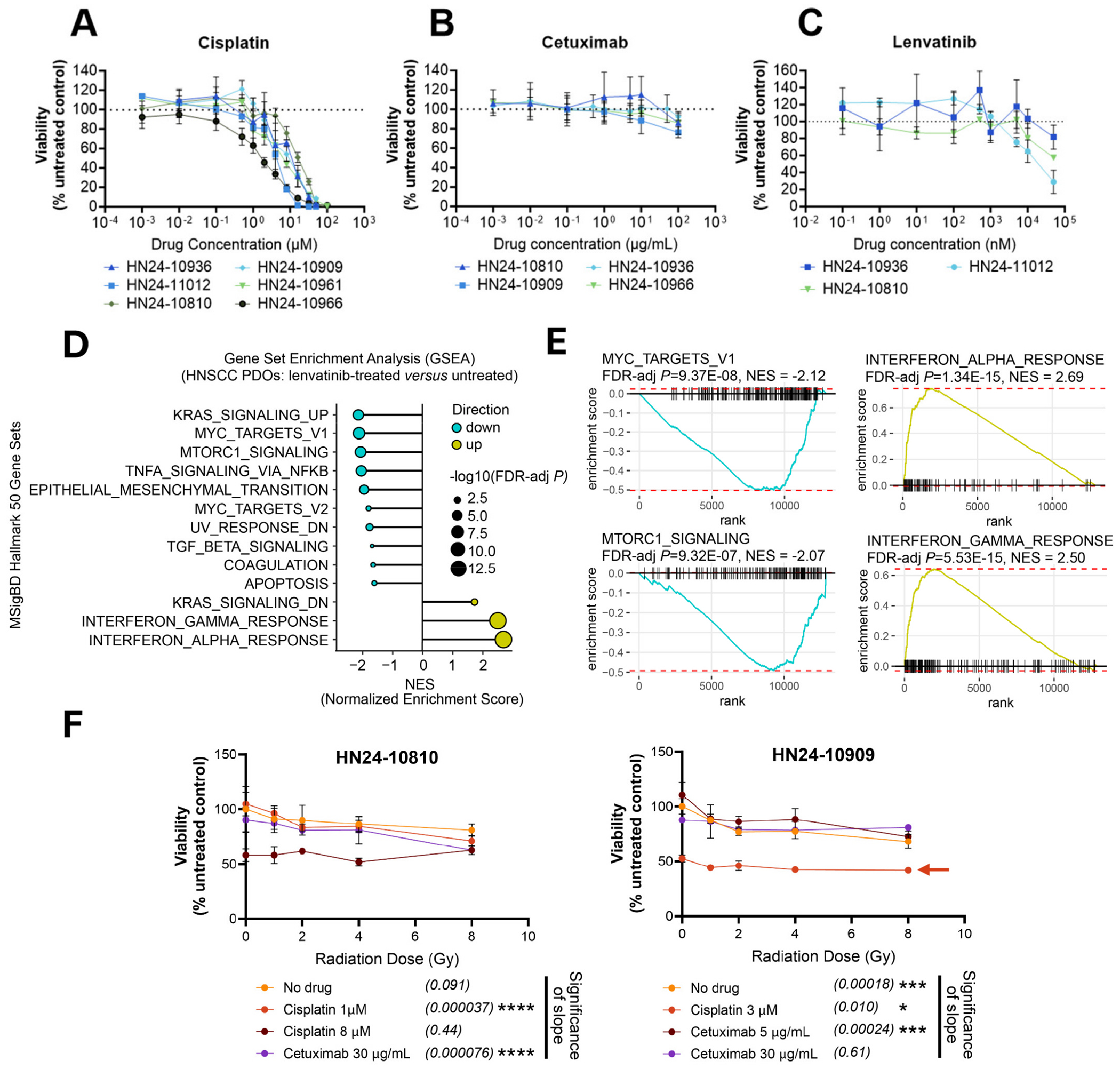
Tumor PDOs demonstrate clinically relevant sensitivity to standard-of-care therapies in HNSCC. (**A-C**) Dose-response curves for cisplatin in **A**, cetuximab in **B**, and lenvatinib in **C** on PDOs from multiple patients. PDOs showed consistent sensitivity to cisplatin (IC50 range: 1–20 μM), while cetuximab and lenvatinib had minimal effects at most tested doses. Viability was measured using the CellTiter-Glo^®^ assay and normalized to untreated control. (**D**) Gene set enrichment analysis (GSEA) of RNAseq gene expression from Lenvatinib (10^2^ nM, 24 h)-treated PDOs (FDR-adjusted *P* < 0.05) from four patients (eight samples total). Top MSigDB Hallmark 50 gene sets (H50) are shown on the row. Dots are colored by direction (NES > 0: upregulated; NES < 0: downregulated), and their size are scaled by −log10(FDR-adjusted p-value). A full list of H50 GSEA results is provided in Table S9. (**E**) GSEA plots showing lenvatinib induced downregulation of MYC targets and OXPHOS, and upregulation of type I/II IFN signaling in HNSCC PDOs. Enrichment scores are shown on *y*-axis, and ranked gene positions are shown on *x*-axis. (**F**) Chemoradiation response in two tumor PDO lines. Data points at “Radiation Dose (Gy) = 0” on the x-axis represents cisplatin alone (1, 3, or 8 μM). For each treatment group, four replicates were included. P-values in parathesis next to each treatment group represent whether the slope of fitted models (indicating radiation dose-dependent cell viability reduction) was significantly different from zero within that group (full statistics are provided in Table S10). Significant slopes indicate that chemoradiation induced stronger viability reduction compared to cisplatin alone (0 Gy). Denotation: * *P* < 0.01, *** *P* < 0.001, **** *P* < 0.0001. Two-sided hypergeometric test was used in **D**. Linear mixed-effects models were used in **F**, with radiation dose as the fixed effect and replicate id as the random effect. P-values shown in **D** and **F** were after multiple comparison adjustments by BH-FDR method.

Building on the preserved histologic and genomic fidelity of PDOs, we next evaluated their drug sensitivity profiles. Stable tumor organoid cultures were treated with cisplatin (chemotherapy), cetuximab (anti-EGFR), lenvatinib (a multiple RTK inhibitor against VEGFR1-3) (Table S7), radiation, and chemoradiation, and measured for cell viability. Cisplatin induced a dose-dependent reduction in viability as assessed by CellTiter-Glo, with IC_50_ values ranging from 1.61 to 15.69 μM across cases (Fig. 4A; Table S8). In contrast, cetuximab showed a minimal effect on PDO viability (Fig. 4B), consistent with literature reporting response to cetuximab in HNSCC is heterogeneous and often limited [47]. Similarly, lenvatinib did not significantly reduce viability across a range of doses, except at very high concentrations, likely reflecting off-target toxicity (Fig. 4C).

Lenvatinib targets angiogenic signaling through VEGF pathway blockade and is being investigated in combination with immunotherapy in ongoing clinical trials. To better understand its biological effects in PDOs, we performed RNAseq on treated and untreated samples from four PDO lines, followed by gene set enrichment analysis (GSEA) (Table S9). Lenvatinib treatment significantly downregulated proliferative and metabolic pathways, including MYC targets, mTORC1 signaling, among others (FDR-adjusted *P* < 0.05; Fig. 4D). In contrast, inflammatory programs, such as interferon alpha and gamma response signatures, were upregulated (Fig. 4E). These findings align with prior reports that RTK inhibitors mediate both anti-proliferative and immune-modulating effects in human tumors [48].

Given that chemoradiation is a standard treatment for locally advanced or recurrent HNSCC, we examined its effects on tumor PDOs to assess their utility as a translational model (Table S10). In HN24-10810 PDO line (Fig. 4F, left), radiation alone (no drug) reduced cell viability (FDR-adjusted *P* = 0.091, by LMMs). Combining radiation with low-dose cisplatin (1 μM) or cetuximab (30 μg/mL) significantly enhanced cell killing (*P* < 0.0001), whereas this effect was not observed with high-dose cisplatin (8 μM; *P* = 0.44). In HN24-10909 (Fig. 4F, right), radiation alone significantly reduced viability (*P* = 0.00018). Cisplatin alone (0 Gy) also reduced viability, and increasing radiation dose to 8 Gy produced an additional 5–10 % reduction (*P* = 0.010; red arrow), indicating an additive chemoradiation effect. A similar radiation dose-dependent viability decline occurred with low-dose cetuximab (5 μg/mL) but not with high-dose cetuximab (30 μg/mL). Patient HN24-10909 received adjuvant chemoradiation following surgery; however, due to the timing of therapy, we could not isolate the clinical benefit of chemoradiation from surgical outcomes. Collectively, these findings show that HNSCC PDOs respond to radiation and chemotherapeutic agents in a treatment- and dose-dependent manner, supporting their potential as a functional platform for testing therapeutic regimens.

## Discussion

We established PDOs from a diverse cohort of HPV-negative HNSCC patients, representing various anatomical sites, tumor T-categories, and demographics. To address the challenge of distinguishing malignant from normal organoids, we created TransferNet-PDO, a novel transfer-learning based CNN that enables rapid and accurate classification of organoid identity at single-cell resolution, achieving high performance across passages and patients (AUC ≥ 0.88). This AI tool facilitates morphological tracking over time and under treatment conditions, which can be adapted for dynamic drug response monitoring, functional screening, and multi-center biobanking. Future iterations incorporating multimodal data [49] (e.g., transcriptomics, proteomics) and histology foundation models [29–34] with fine tuning may further enhance classification and support therapeutic prediction.

Our platform preserves genomic stability and clonal architecture across serial passages, supporting PDOs as reliable models for personalized therapy. While prior studies have reported genomic shifts in culture [18], our focus on short-term passages (<12; 3 months) retained key genomic features, with ongoing monitoring to assess long-term stability. Short- and long-term PDOs provide complementary strengths for translational research. Short-term cultures can function as patient “avatars” for real-time outcome prediction and monitoring under treatment, while long-term cultures enable modeling of tumor evolution and resistance through large-scale drug screening.

Drug sensitivity testing highlights the translational relevance of our PDO models. Cisplatin reduced PDO viability in a dose-dependent manner, while cetuximab and lenvatinib did not eliminate tumor organoids, aligning with known clinical scenarios. As an NAM platform, PDOs offer the potential to model therapy resistance, such as clonal selection in the TME, which may advance our understanding of treatment failure. Incorporating PDOs from longitudinal tumors at recurrence or progression may further strengthen this model in interrogating both preexisting and acquired resistant clones.

While multiple PDO technologies exist, the constitutive PDO system offers distinct advantages for genetic and pharmacologic manipulation, allowing precise control over specific cell types [50]. HNSCC PDOs can be engineered to express defined alterations (e.g. NOTCH1/2 mutations [51]) to study drug-resistant subclones, with potential to inform rational combination strategies involving novel small-molecule or immune checkpoint inhibitors [52]. Moreover, the platform provides a tractable system to evaluate CAR T cell efficacy [53,54] in a personalized context, including antigen targeting, cytotoxicity, and mechanisms of immune evasion.

Our study has limitations. The patient cohort, while diverse, remains modest in size, which may limit generalizability across the broader HNSCC population. For patients who underwent surgery without further treatment, direct correlations between PDO responses and clinical outcomes will require long-term follow-up upon progression. With respect to functional testing, cisplatin was evaluated as a proof-of-concept agent, but its clinical role in HNSCC is largely as a radiosensitizer. While we included chemoradiation assays to address this, future studies incorporating fractionated radiation with concurrent cisplatin, as well as broader evaluation of combination regimens, will be needed to better mirror standard-of-care therapy in the PDO system. Expanding our cohort to include HPV-positive oropharyngeal cancers (OPCs) will be an important next step to broaden the applicability of this platform.

Technical and biological considerations further highlight opportunities for refinement. We selectively applied Nutlin-3 to suppress p53 wild-type normal epithelial overgrowth [14], however this strategy may also reduce intratumoral heterogeneity by eliminating tumor-normal interactions present in native tissue. Thus, potential bias in cellular composition should be carefully monitored by DNA sequencing with orthogonal validation (e.g., single-cell RNAseq). Trypsin dissociation may also alter cell-surface glycoproteins and affect long-term genomic stability of PDOs [18]. Alternative approaches (e.g., collagenase/dispase digestion) may help preserve native surface features and mitigate this risk. In addition, our current PDO system is primarily epithelial, and does not fully capture the extracellular matrix (ECM) dynamics or crosstalk between the tumor and surrounding tissue that collectively shape tumor behavior and therapy response. Future directions include tumor-immune-stroma assembloids, TME-mimicking ECM scaffolds [55], and hypoxia culture conditions [56], which may provide more physiologically relevant models. Finally, our AI classifier, while robust, relies primarily on nuclear and regional features and may underutilize cytoplasmic information. Advanced 3D imaging or single-organoid microengineered devices [53] could enable real-time, label-free tracking of tumor growth and drug response.

This work opens several translational avenues. Longitudinal PDOs from sequential patient samples could track tumor evolution and resistance, supporting real-time adaptation of therapeutic strategies, for instance, identifying when to transition from immunotherapy to chemotherapy, or when chemotherapy should be added prior to clinical progression in recurrent HNSCC. Furthermore, assembloids incorporating tumor, immune, and stromal components may more faithfully model the TME and enable identification of patient-specific immunotherapy biomarkers or functional validation of computationally predicted drug combinations. Pairing these models with AI tools trained on multi-omic PDO data provides a path toward scalable, high-throughput therapeutic prioritization. Lastly, our finding that PDOs can be generated from small tissue inputs demonstrate feasibility for biopsies, particularly in recurrent, inoperable, or metastatic cases, where improved understanding of drug sensitivity and resistance evolution could be transformative.

Beyond HNSCC, our PDO platform has broader implications for precision oncology. The approach is adaptable to other heterogeneous solid tumors, such as pancreatic and lung cancers, where predictive biomarkers remain elusive. Integrating PDOs with AI-driven analytics offers a generalizable framework for drug repurposing, combination therapy design, and biomarker discovery across cancer types on rapid translation of personalized therapies from bench to bedside.

## Conclusion

We established a clinically relevant PDO platform for HPV-negative HNSCC that preserves tumor fidelity, enables drug response modeling, and supports AI-driven classification. These integrated tools provide a foundation for developing complex assembloids and personalized therapeutic strategies in head and neck cancer.

## Supplementary Material

supp1

supp2

## Figures and Tables

**Table 1:** Clinical and demographic characteristics of HNSCC patients.

Study ID	PID	Age in years	Tumor Location	T-category (path)	Type of Tumor	Grade	Tumor P53 IHC status	Organoids status	Nutlin selection	Organoid max passage no. banked
HN23-10728	P001	47	Larynx	T3	Primary	POOR	Unknown	Failed	NA^a^	NA^1^
HN23-10730	P002	75	Larynx	T3	Primary	POOR	Positive	Tumor	No	12
HN23-10750	P003	75	Oral cavity (floor of mouth)	T3	Primary	POOR	Unknown	Failed	NA^1^	NA^1^
HN23-10752	P004	58	Larynx	T4A	Primary	MOD	Positive	Normal	No	9^b^
HN24-10791	P005	62	Larynx	T4	Unknown	MOD	Positive	Normal	No	5^2^
HN24-10798	P006	76	Tongue	T2	Unknown	WELL	Unknown	Failed	NA^a^	NA^1^
HN24-10810	P007	54	Oral cavity (floor of mouth)	T2	Primary	MOD	Negative	Tumor	No	19^d^
HN24-10831	P008	64	Larynx	T3	Primary	MOD	Positive	Normal	No	6^2^
HN24-10856	P009	81	Oral cavity (gum)	TX	Recurrence	MOD	Positive	Tumor	No	8
HN24-10900	P010	52	Oropharynx	T3	Recurrence	Unknown	Positive	Tumor	No	6
HN24-10909	P011	58	Tongue	T3	Primary	MOD	Positive	Tumor	No	12
HN24-10936	P012	82	Oral cavity (cheek)	T2	Primary	MOD	Unknown	Tumor	Both^f^	6
HN24-10942	P013	54	Larynx	NE^e^	Primary	MOD	Positive	Normal	No	3^2^
HN24-10961	P014	59	Larynx	T4a	Primary	MOD	Positive	Tumor	Yes	7
HN24-10966	P015	44	Tongue	T3	Primary	WELL	Negative	Tumor	Both^f^	16^4^
HN24-10970	P016	78	Larynx	T4a	Primary	POOR	Unknown	Failed	NA^1^	NA^1^
HN24-10981	P017	68	Larynx	T4a	Recurrence	MOD	Unknown	Dead tissue	NA^1^	NA^1^
HN24-10988	P018	57	Larynx	T3	Primary	MOD	Negative	Normal	No	2^2^
HN24-11012	P019	64	Oral cavity	T4a	Second primary	MOD	Negative	Tumor	No	6
HN24-11042	P020	62	Larynx	T4a	Second primary	MOD	Positive	Tumor	No	2^c^
HN24-11074	P021	64	Oral cavity (buccal mucosa)	T4a	Primary	MOD	Positive	Tumor	Both^f^	3^4^
HN25-11105	P022	56	Oral cavity (buccal mucosa)	T4A	Primary	POOR	Negative	Tumor	No	4^3^
HN25-11113	P023	68	Oral cavity (floor of mouth)	T4a	Primary	MOD	Unknown	Tumor	Yes	5
HN25-11122	P024	75	Tongue	T4a	Primary	MOD	Positive	Tumor	Both^f^	9^4^

Footnotes:

a NA = Not Applicable.

b Banked after normal PDOs outgrew normal PDOs.

c Banked early due to growth rate was slow.

d Active in culture. Passage number increasing at time of manuscript submission.

e NE = Not Evaluable. Biopsy only, therefore no path stage was evaluated per AJCC guidelines.

f Both = both selected and unselected cultures banked.

## Appendix A. Supplementary data

Supplementary data to this article can be found online at https://doi.org/10.1016/j.oraloncology.2025.107742.
